# Brain structure–function coupling associated with cognitive impairment in cerebral small vessel disease

**DOI:** 10.3389/fnins.2023.1163274

**Published:** 2023-06-06

**Authors:** Na Wang, Changhu Liang, Xinyue Zhang, Chaofan Sui, Yian Gao, Lingfei Guo, Hongwei Wen

**Affiliations:** ^1^Department of Radiology, Shandong Provincial Hospital Affiliated to Shandong First Medical University, Jinan, Shandong, China; ^2^Key Laboratory of Cognition and Personality (Ministry of Education), Faculty of Psychology, Southwest University, Chongqing, China

**Keywords:** cerebral small vessel disease, cognitive impairment, structure–function coupling, neurovascular coupling, multimodal magnetic resonance imaging

## Abstract

Cerebral small vessel disease (CSVD) is a common chronic and progressive disease that can lead to mental and cognitive impairment. Damage to brain structure and function may play an important role in the neuropsychiatric disorders of patients with CSVD. Increasing evidence suggests that functional changes are accompanied by structural changes in corresponding brain regions. Thus, normal structure–function coupling is essential for optimal brain performance, and disrupted structure–function coupling can be found in many neurological and psychiatric disorders. To date, most studies on patients with CSVD have focused on separate structures or functions, including reductions in white matter volume and blood flow, which lead to cognitive dysfunction. However, there are few studies on brain structure–function coupling in patients with CSVD. In recent years, with the rapid development of multilevel (voxel-wise, neurovascular, regional level, and network level) brain structure–functional coupling analysis methods based on multimodal magnetic resonance imaging (MRI), new evidence has been provided to reveal the correlation between brain function and structural abnormalities and cognitive impairment. Therefore, studying brain structure–function coupling has a potential significance in the exploration and elucidation of the neurobiological mechanism of cognitive impairment in patients with CSVD. This article mainly describes the currently popular brain structure–function coupling analysis technology based on multimodal MRI and the important research progress of these coupling technologies on CSVD and cognitive impairment to provide a perspective for the study of the pathogenesis and early diagnosis of CSVD.

## 1. Introduction

Cerebral small vessel disease (CSVD) is a general term for intracranial vascular (including arteriolar, arteriolar, capillary, and venular) diseases based on a variety of pathological and neurological processes and refers to syndromes with different clinical manifestations and neuroimaging features caused by structural changes in blood vessels and brain parenchyma (Cuadrado-Godia et al., 2018; Li et al., 2018). CSVD is one of the major diseases affecting cognitive function and extremity function. According to previous studies, the main clinical manifestations of CSVD include cognitive decline, stroke, mental disorders, dementia, urinary incontinence, and abnormal gait (Li et al., 2018; Chojdak-Lukasiewicz et al., 2021). Researchers have shown that CSVD has become the main cause of stroke, depression, cognitive impairment, dementia, and gait disturbance in elderly individuals (Rost and Etherton, 2020). The use of advanced magnetic resonance imaging (MRI) technology can detect cerebrovascular abnormalities before the disease appears (Zabetian-Targhi et al., 2019). Brain imaging biomarkers for CSVD include white matter hyperintensities, cerebral microbleeds, recent small subcortical infarcts, lacunae, brain atrophy, and enlarged perivascular spaces (Cuadrado-Godia et al., 2018; Li et al., 2018).

MRI can detect brain structure and function. It is well known that brain structure and function are closely related. The coupling of structural imaging and functional imaging has become an increasingly important research topic in modern neuroimaging. The brain structure can provide the skeleton for functional mechanisms, and the integration of functional and structural information can help us better understand communication in the brain. Thus, changes in brain function may lead to changes in the gray or white matter and vice versa. However, most studies have only examined functional or anatomical changes in isolation. In recent years, with the rapid development of multimodal MRI technology, researchers have carried out a large number of neuroimaging studies on the structural and functional damage to the brain caused by CSVD (Liu et al., 2019; Meng et al., 2022). Studies have shown that changes in brain function are often accompanied by changes in the structure of the corresponding brain regions. For example, the persistence, intensity, and spatial statistics of brain activities in the resting state are usually limited by the anatomical structure of the brain (Honey et al., 2009). Thus, normal structure–function coupling is essential for the brain, and disruptions in structure–function coupling can be found in many neurological and psychiatric disorders (Zhang X. et al., 2022). Therefore, the exploration of brain structure–function coupling is more significant for elucidating the neurobiological mechanisms of cognitive and mental impairment in CSVD.

Brain structure–function coupling exists at various levels, including voxel, neurovascular, regional, and network levels. Neurovascular coupling (NVC) is a tight regional and temporal coupling in the brain, which means that brain regions with stronger connectivity tend to have more frequent neuronal activity and more energy expenditure, resulting in increased cerebral blood flow (CBF) (Li P. et al., 2021). The dysfunction of structure–function coupling of NVC may have great significance in the neuropathological mechanism of CSVD. As an important mechanism to achieve autonomic regulation of CBF, NVC can ensure appropriate blood supply to maintain basic physiological activities such as respiration, blood pressure, and endocrine function and carry out a series of advanced cognitive functions such as memory, learning, and emotion. NVC is a complex process that involves the coordination and feedback loops of multiple cells. The basic structure for the realization of NVC function is the neurovascular unit, which is composed of neurons, astrocytes, vascular smooth muscle cells/pericytes, and Virchow–Robin space (Kaplan et al., 2020). In the field of neuroimaging, NVC is the physiological basis of functional MRI (Whittaker et al., 2015). In terms of pathophysiology, disruption of its function is one of the important pathogeneses of a variety of ischemic nervous system diseases, including CSVD (Sutherland et al., 2017). However, numerous current NVC studies mainly focus on temporal and regional associations between neural activity and CBF responses but ignore major structural factors related to NVC, including the anatomical structure of cerebral vasculature and organ structure regulating CBF (Phillips et al., 2016). In addition to functional neuroimaging, future research should consider further introducing structural neuroimaging features to assess the NVC and highlight the related alterations in the NVC underlying CSVD.

To date, most studies on patients with CSVD have focused on separate structural or functional abnormalities but have ignored the important relationship between them, which may be highly related to cognitive dysfunction. Although structure–function coupling information from neuroimaging techniques is essential for understanding abnormal changes in brain diseases, there are few studies on brain structure–function coupling in patients with CSVD. Defining the significance of coexisting structural and functional deficits can provide specific insights into the changes that occur in the brains of CSVD patients.

The aim of this review is to describe a series of useful structure–function coupling analysis methods based on multimodal MRI and the research progress of brain structure–function coupling related to cognitive impairment and to provide a new perspective for the study of CSVD.

## 2. Voxel-wise structure–function coupling

There are two commonly used voxel-wise structure–function coupling approaches: one is the coupling of resting-state functional MRI (rs-fMRI) parameters and gray matter (GM) morphological parameters, and the other is the coupling of rs-fMRI parameters and diffusion tensor imaging (DTI)-based white matter (WM) microstructural parameters.

In the first approach, Kang et al. (2022) combined regional homogeneity (ReHo) and voxel-based morphometry (VBM) features and calculated the ratio of ReHo to GM volume (GMV) to detect altered voxel-wise structure–function coupling and its importance in predicting radiation encephalopathy (RE) in patients with nasopharyngeal carcinoma (NPC). They found that compared with the preradiotherapy group, patients in the postradiotherapy group showed lower ReHo/VBM coupling values in the bilateral medial temporal lobes, indicating that ReHo/VBM may be a novel effective neuroimaging metric that reflects the neural mechanism underlying cognitive impairment caused by RE in patients with NPC. Gray et al. (2020) performed a coordinator-based meta-analysis including VBM studies and resting-state voxel-based pathophysiology (VBP) studies (including glucose metabolism, cerebral blood flow (CBF), ReHo, amplitude of low-frequency fluctuation (ALFF), and fractional ALFF) to investigate spatially convergent structural and functional abnormalities in major depressive disorder (MDD) patients and identified that the regions of significant convergence include the right middle occipital gyrus, inferior temporal gyrus, amygdala/putamen and left hippocampus, subgenual cingulate cortex, and retrosplenial cortex, which may contribute to the cognitive and recall memory deficits often seen in MDD patients. A study on schizophrenia showed that the ALFF value in the hippocampus of schizophrenia patients was significantly different from the GMV measurements, and this difference may be due to cognitive and emotional dysfunction caused by hippocampal damage, which is related to the production of hallucinations in schizophrenia patients (Zhao et al., 2017). These studies suggest that the brain structural defect and functional defect are related to each other, and the differences in this correlation indicated that the structure and function relationships of the brain regions have changed.

In the second approach, Tan et al. combined DTI and rs-fMRI and found that patients with classical trigeminal neuralgia had high fractional anisotropy (FA) and low ReHo in the right hippocampus, and the ReHo and FA values were positively correlated with the Montreal Cognitive Assessment scale score. This indicated that there is a clear correlation between cognitive dysfunction and hippocampal functional and structural dysfunction in classical trigeminal neuralgia patients. Strong evidence has been provided showing that pain depends on the activation of mechanisms in higher brain centers that control emotions and generate emotional recognition (Tan et al., 2022). In addition, some researchers have used fMRI and DTI techniques to propose a skeleton-based method for WM functional analysis, which achieves voxel-based function–structure coupling by projecting fMRI signals onto the skeleton in WM. The researchers' study showed that local WM regions (frontotemporal tracts including the right anterior corona radiata, the orbitofrontal region, the hippocampus, the anterior limb of internal capsule, and the genu of corpus callosum) in patients with schizophrenia demonstrated increased amplitude of low-frequency fluctuations (SWALFF) and decreased FA. The correlation between FA changes and SWALFF changes was negative in schizophrenia patients but positive in healthy controls (Jiang et al., 2021). Thus, hard evidence was provided to support the hypothesis that structure–function associations may be one of the underlying mechanisms of impaired connectivity in schizophrenia patients.

Recently, to further develop the voxel-wise coupling analysis method, Hu et al. proposed a new coupling method called principal-component-based intermodal coupling (pIMCo) (Hu et al., 2022). The method uses local covariance decomposition to define a symmetric, voxel-wise coupling coefficient that holds for two or more modal parameters. Hu et al. used this approach to investigate the coupling among CBF, ALFF, and ReHo, demonstrating that coupling is spatially heterogeneous, varies with age and sex in neural development, and reveals patterns that are absent in individual patterns. This new approach provides a new perspective for summarizing the overall covariance structure between more than two modalities.

## 3. Brain network structural-functional connectivity coupling

The graph theory of brain networks conceptualizes the brain as a network that achieves functional performance through the interaction among various brain regions through structural connectivity (SC) or functional connectivity (FC) (Shah et al., 2018). The brain network can be assessed by structural and functional neuroimaging data. CSVD can affect SC and FC by damaging the integrity of the nodes in the network and the connections between them, thus disrupting the effective communication of the whole-brain network and leading to different degrees of cognitive impairment. It has been shown that the coupling of SC and FC based on multimodal MRI allows the detection of brain network disruption, which is more sensitive than any single modality (Kong et al., 2021). However, the changes in SC-FC coupling of brain networks at the system level in CSVD patients are still unclear and need further investigation.

Studies have shown significantly altered SC and FC between hippocampal cognitive and emotional subregions and cortical areas in patients with cognitive impairment, which is manifested as an increase in the early stage of the disease, possibly due to compensatory mechanisms (Liang et al., 2020; Xu et al., 2021). Filippi et al. found that hippocampal SC changes were greater than FC changes in patients with Alzheimer's disease and amnestic mild cognitive impairment (MCI), indicating that reduced SC may precede FC changes (Filippi et al., 2020). Other studies have found that subcortical vascular MCI has significant SC and FC reductions in the brain, especially in the brain regions connected to the hippocampus, which may be related to cognitive impairment in patients with this disease, while the FC of hippocampal subregions and the posterior cingulate cortex is enhanced. Since the functional interaction between the hippocampus and the posterior cingulate cortex is related to episodic memory, this interaction can be interpreted as a compensatory mechanism for memory impairment; the increased structural association between the inferior collicularis of the hippocampus and the pole part of the superior temporal gyrus in amnestic MCI may be related to depressive symptoms, such as difficulties in processing facial emotions in patients (Xu et al., 2021). These findings suggest that different brain regions may have specific relationships with depressive symptoms and/or cognitive impairment. In addition, another study found that the Papez circuit, including the amygdala, ipsilateral hippocampus, caudal anterior cingulate gyrus, and thalamus, had significant GM atrophy, and the directional functional connection between the cingulate gyrus and the bilateral hippocampus within the Papez circuit was also altered in stroke patients. These alterations in effective connectivity have been associated with cognitive function after cerebrovascular events (Yan et al., 2022). A previous study also found selectively abnormal SC-FC coupling in subcortical vascular MCI, that is, SC-FC coupling is preserved at the whole-brain level but is increased in the dorsal attention module and reduced in the ventral attention module (Ma et al., 2021). These findings motivate us to obtain a better understanding of the underlying mechanisms of vascular cognitive impairment and to explore novel therapeutic targets that could reduce the cognitive burden.

## 4. Structural and functional neurovascular coupling

The tight coupling between neuronal activity and CBF is called NVC (Li L. et al., 2021). As an important mechanism regulating CBF, NVC can deliver oxygen and nutrients to regions in a timely manner according to the requirements of brain activity to maintain the homeostasis of the brain environment. Cerebral small vessels play a critical role in the automatic regulation of the brain, which mainly depends on the normal operation of the NVC. Impairment of the NVC mechanism can lead to ischemia and hypoxia of small vessels located at the end of cerebral microcirculation, resulting in the failure of the local brain to respond to neuronal signal transduction, thus causing or aggravating small vessel diseases and even cognitive dysfunction and dementia. In recent years, NVC has been widely studied for its key role in the automatic regulation of CBF in the brain. Therefore, understanding the regulatory mechanism of NVC in CSVD is of great significance for preventing the further development of CSVD and improving cerebral ischemia and cognitive dysfunction.

There is increasing evidence that impaired NVC is present in many patients with CSVD. Increased microvascular damage and a decrease in the number of microvessels in the hippocampus and neocortex can cause a decrease in vasodilator reserve, leading to impaired NVC function, which is associated with cognitive decline (Phillips et al., 2016; Kirschen et al., 2018), and the decline in cognitive function can cause a decrease in CBF. Previous animal experiments demonstrated reduced blood flow, oxygenation, and NVC in the hippocampus of mice with cerebrovascular dysfunction (Li P. et al., 2021). At the same time, the hippocampus was more susceptible to hypoxic damage under pathological conditions that led to impaired NVC (Zhang H. et al., 2022). Studies have shown that structural and functional changes in neurovascular aspects may underlie cognitive decline in numerous pathological states (Segarra et al., 2019). Some researchers have used mathematical dynamic multivariate and autoregressive models, in which the changes in critical closing pressure and the resistance area product are considered to provide selective indicators of metabolic and myogenic cerebrovascular regulation, respectively, in the hope of providing insights into the mechanisms of NVC from a structural perspective (Phillips et al., 2016). Previous studies of NVC have mainly focused on the coupling of the functional indicators ALFF and CBF and have not involved the content of the structural NVC. Therefore, further studies are needed to clarify the specific role of structure–function NVC in CSVD.

## 5. Hippocampal structure–function coupling

As an important anatomical structure, the hippocampus is of great significance in the pathogenesis of neuropsychiatric diseases. Neuropsychiatric disorders caused by CSVD can lead to structural and functional damage to the hippocampus in many ways. Indeed, hippocampal neuronal loss and atrophy, regional cerebral blood volume reduction, and hippocampal microinfarction have been observed in patients with CSVD (Perosa et al., 2020). Correspondingly, when the structure and function of the hippocampus are damaged (especially if both exist at the same time), the cognitive, emotional, and motor abilities of the patient are greatly affected (van Norden et al., 2012; Wu et al., 2022). Therefore, the study of structure–function coupling specifically targeting the hippocampus may be more meaningful for CSVD.

At present, structure–function coupling analysis methods and research specific to the hippocampal region are developing continuously. Recently, Bayrak et al. isolated the hippocampus and investigated its structure–function coupling using the twin setup of the Human Connectome Project with multimodal neuroimaging (Bayrak et al., 2022). To measure structure–function coupling, they assessed the degree of spatial overlap between hippocampal microstructure intensity covariance and FC measures at the vertices of each subfield. In addition, they examined the shared organization of hippocampal function and structure to characterize the spatial covariation in structure–function associations along the genetic hippocampal organization axis. They used the connectivity gradient method rather than the network decomposition method because the connectivity gradient method positions brain regions in a continuous manner according to their functional connectome patterns, whereas network decomposition draws sharp boundaries for brain regions (Krienen and Sherwood, 2016). The coupling between structure and function was found to be highest in the medial/posterior part of the hippocampal subfields but not in the anterior part. In addition, hippocampal structure–function coupling showed covariation with intrinsic structural and functional axes, with the posterior regions having major structural and functional connections to unimodal cortical regions, whereas anterior regions are linked to the transmodal cortex. The study by Bayrak et al. provided an important step toward a better understanding of how the anatomy of the hippocampus supports its versatile and unique functions.

## 6. Conclusion and outlook

In summary, the above structure–function coupling methods, including voxel-wise structure–function coupling, SC-FC coupling, and NVC, are of great significance for CSVD, but the current research is not perfect, and most of them only focus on independent structural or functional aspects. In addition, although multimodal MRI has been widely used in studies of CSVD, some issues remain. For instance, the mechanisms leading to cognitive impairment are complex and may be the result of a combination of factors other than CSVD alone (Lawrence et al., 2013). Second, since each CSVD patient usually has a different combination of MRI markers and severity, each single MRI marker may be difficult to use to predict individual clinical outcome (Lee et al., 2021). Disruptions in the brain structure and function have been widely recognized to be associated with cognitive decline in CSVD, but the neural mechanisms remain unclear (Xu et al., 2021). Different MRI examination methods have their own advantages and disadvantages; however, if the structural imaging and functional imaging of the brain are coupled and the anatomical information and functional information are integrated, it will be helpful for the early diagnosis and prediction of CSVD progression.

**Figure 1:**
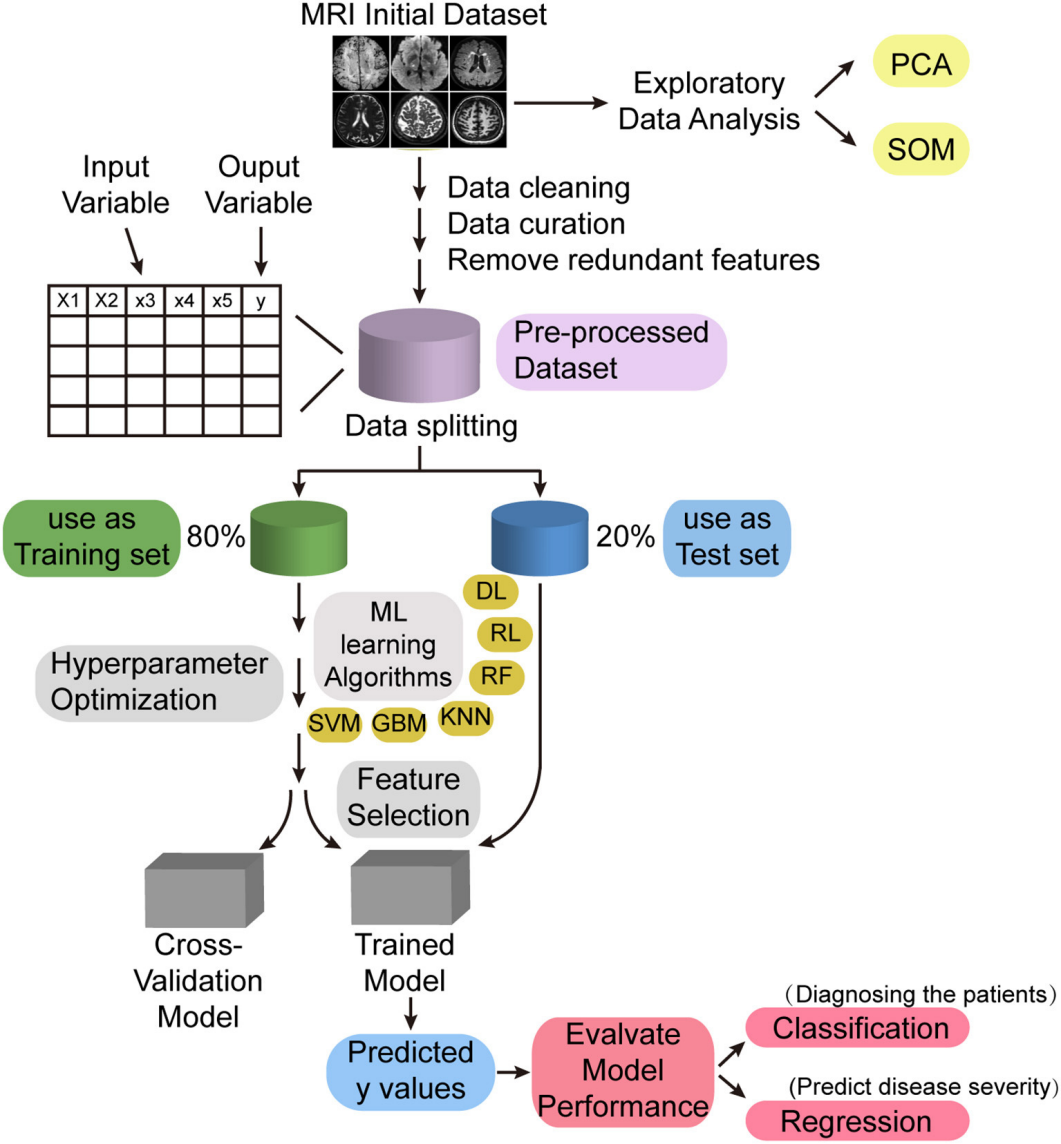
Machine learning model for intelligent diagnosis of diseases. PCA, principal component analysis; SOM, self-organizing map; ML, machine learning; DL, deep learning; RL, reinforcement learning; RF, random forest; KNN, K-nearest neighbor; GBM, gradient boosting machine; SVM, support vector machine.

In addition, advances in machine learning have led to the development of artificial intelligence algorithms that can aid in the diagnosis of diseases based on MRI for detection at the preclinical stage. Using machine learning techniques, Uysal and Ozturk, successfully differentiated patients with cognitive impairment, patients with Alzheimer's disease, and normal subjects by hippocampal volume information (Uysal and Ozturk, 2020); Sarwar et al. also demonstrated through machine learning that the structure–function coupling in the human brain is very tight (Sarwar et al., 2021). Therefore, as a novel neuroimaging feature, brain structure–function coupling can be combined with machine learning technology to establish a computer-aided diagnostic system based on multimodal MRI data (Figure 1), and the structure–function coupling features highly correlated with CSVD and cognitive impairment can be mined through machine learning methods, which can help clinicians diagnose patients with CSVD and predict the severity of this disease.

Therefore, with the continuous development of multimodal MRI fusion analysis technology, brain structure–function coupling will become a hot spot in the study of CSVD in the future.

## Author contributions

NW and CL wrote the main manuscript text. XZ, CS, and YG prepared the imaging data. LG and HW revised the main manuscript text. All authors reviewed the manuscript. All authors contributed to the article and approved the submitted version.
